# Trk agonist drugs rescue noise-induced hidden hearing loss

**DOI:** 10.1172/jci.insight.142572

**Published:** 2021-02-08

**Authors:** Katharine A. Fernandez, Takahisa Watabe, Mingjie Tong, Xiankai Meng, Kohsuke Tani, Sharon G. Kujawa, Albert S.B. Edge

**Affiliations:** 1 Department of Otolaryngology, Harvard Medical School, Boston, Massachusetts, USA.; 2 Eaton-Peabody Laboratories, Massachusetts Eye and Ear, Boston, Massachusetts, USA.; 3 Program in Speech and Hearing Bioscience and Technology, Harvard Medical School, Boston, Massachusetts, USA.; 4 Harvard Stem Cell Institute, Cambridge, Massachusetts, USA.

**Keywords:** Otology, Neurodegeneration, Synapses

## Abstract

TrkB agonist drugs are shown here to have a significant effect on the regeneration of afferent cochlear synapses after noise-induced synaptopathy. The effects were consistent with regeneration of cochlear synapses that we observed in vitro after synaptic loss due to kainic acid–induced glutamate toxicity and were elicited by administration of TrkB agonists, amitriptyline, and 7,8-dihydroxyflavone, directly into the cochlea via the posterior semicircular canal 48 hours after exposure to noise. Synaptic counts at the inner hair cell and wave 1 amplitudes in the auditory brainstem response (ABR) were partially restored 2 weeks after drug treatment. Effects of amitriptyline on wave 1 amplitude and afferent auditory synapse numbers in noise-exposed ears after systemic (as opposed to local) delivery were profound and long-lasting; synapses in the treated animals remained intact 1 year after the treatment. However, the effect of systemically delivered amitriptyline on synaptic rescue was dependent on dose and the time window of administration: it was only effective when given before noise exposure at the highest injected dose. The long-lasting effect and the efficacy of postexposure treatment indicate a potential broad application for the treatment of synaptopathy, which often goes undetected until well after the original damaging exposures.

## Graphical abstract

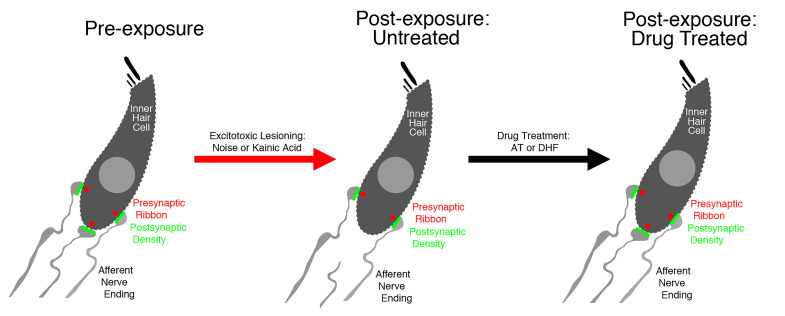

## Introduction

Sensorineural hearing loss results from pathology of the inner ear, and its primary cause can reside in sensory hair cells or afferent neurons (1–7). However, work in animal models of noise- and age-related hearing loss has revealed that loss of synapses between the two is commonly the earliest finding (2, 5). This type of damage has been referred to as hidden hearing loss because its presence is not captured by the threshold audiogram, the standard clinical assay of hearing loss. Although synaptic loss is not revealed by threshold measures until near total, it can be detected as a decline in the suprathreshold amplitudes of neural responses (2, 5, 8).

Neurotrophins have been tested for activity in ameliorating noise– and toxin-induced damage to the neurons of the inner ear (4, 9–12). These molecules have numerous functions in neurons, including support for neural survival, outgrowth of neurites, and both guidance and synapse formation. While neurotrophins have been extensively tested as therapeutic agents, the difficulty in targeting therapeutic levels of proteins to the brain or peripheral nerves has hampered their use (13). In contrast, small molecules can reach target tissues in the nervous system, with demonstrated protective and regenerative effects (14).

Here, we examined the potential of small molecule Trk receptor agonists to act in a similar fashion as neurotrophins when applied to the cochlea. We hypothesized that the small molecules would confer an advantage of easier delivery to the fluid-filled compartments of the cochlea. Since the chemical structure and properties of small molecules have an effect on outcome, we chose 2 structurally unrelated small molecule agonists of the TrkB receptor (15–17), amitriptyline (AT) and 7,8-dihydroxyflavone (DHF), for testing for protection or regeneration of cochlear neural function caused by synaptopathic noise exposure. Using parallel in vitro and in vivo approaches, we characterized protection or rescue from cochlear synapse loss/deafferentation in the mouse cochlea. We found that TrkB agonists were strikingly effective in protection and restoration of synapses and neural function in noise-exposed ears.

## Results

### AT and DHF increased cochlear synaptic regeneration in vitro.

**Figure 1:**
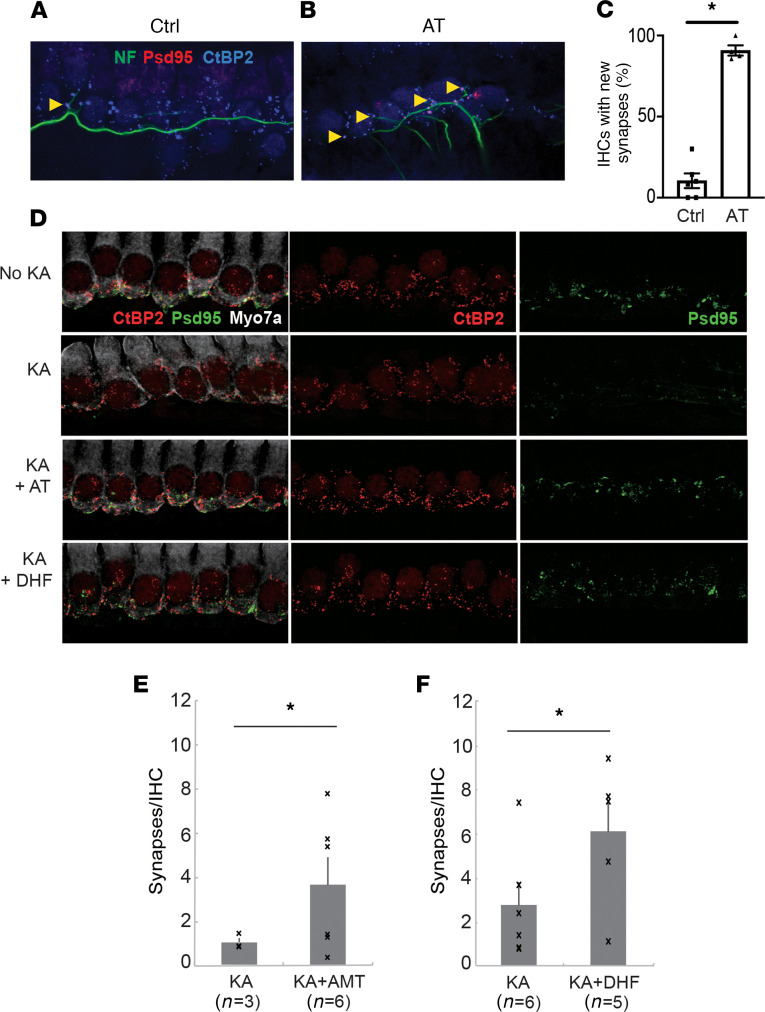
AT and DHF act as TrkB agonists in SGN. (**A** and **B**) Isolated SGNs and denervated organ of Corti were cocultured in the absence (**A**) or presence (**B**) of 0.5 μM AT. After 6 days in culture, explants were fixed and immunostained with antibodies against neurofilament (NF, green), CtBP2 (blue), and Psd95 (red), and confocal images were obtained in the inner hair cell region. (**C**) Juxtapositions of hair cell ribbons and afferent endings were identified by CtBP2/Psd95 puncta (yellow arrowheads in **A** and **B**) and counted, indicating a significant increase in percentage of juxtaposed CtBP2/Psd-95 puncta at inner hair cells after AT treatment. Data are represented as mean ± SEM (*n* = 6 for Ctrl and 4 for AT); Student’s *t* test; *P < 0.05. (**D**) Cochlear explants were cultured with (KA) or without (no KA) exposure to kainic acid (0.4 mM) for 1 hour followed by treatment with culture medium with 0.1 μM AT (KA + AT) or 0.5 μM DHF (KA + DHF). The cultures were immunostained for CtBP2 (red) and Psd95 (green) after 48 hours. (**E** and **F**) AT (**E**) and DHF (**F**) induced significant synaptic regeneration. Data are represented as mean ± SEM (*n* = 3 for no KA, 3 for KA, and 6 for KA + AT in **E**; *n* = 4 for no KA, 6 for KA, and 5 for KA + DHF in **F**; 2-tailed, unpaired Student’s *t* test; *P < 0.05.

To assess synapse formation in a culture system, in which spiral ganglion neurons (SGNs) were cocultured with deafferented organ of Corti (18, 19), the cocultures were treated with 0.5 μM AT or maintained as controls. Six days later, IHC demonstrated that cochlear afferent synapses had regenerated in the cultures (Figure 1, A and B). Quantitative measurement indicated a significant increase in synaptic regeneration in the AT treated cultures (Figure 1C, *P* < 0.05).

We adapted a rat model of excitatory cochlear synaptopathy (20) to the mouse explants to assess the regenerative effect of the drug on cochlear synapses. In this model, cochlear explants, consisting of hair cells and attached SGNs, are exposed to selective ionotropic glutamate receptor agonist, kainic acid, to mimic glutamate toxicity, which is the excessive release of glutamate into the synaptic cleft with attendant damage to the terminal processes of SGNs (21, 22). Synapses were detected by the occurrence of CtBP2-expressing synaptic ribbons and Psd95^+^ postsynaptic densities. Damage was extensive after 1 hour of treatment with kainic acid, and a small amount of reinnervation of hair cells by the peripheral processes of the neurons was apparent after 24 hours in the absence of drugs (Figure 1D). Addition of 0.1 μM AT (Figure 1E) or 0.5 μM DHF (Figure 1F) to the in vitro synaptogenesis assay resulted in a significant increase in juxtaposed ribbons and Psd95^+^ neural endings relative to untreated samples.

### AT rescued synapses and auditory function in vivo.

Prior studies in adult CBA/CaJ mice have demonstrated that exposure to a 2-hour, 100 dB sound pressure level (SPL) octave band noise (8–16 kHz) results in a large but reversible threshold shift, as evidenced by distortion product otoacoustic emission (DPOAE) and auditory brainstem response (ABR) wave 1 measures. The exposure causes no acute loss of inner or outer hair cells, but it initiates an immediate and largely permanent loss of synapses between auditory nerve terminals and cochlear inner hair cells (IHCs) in mid- to high-frequency locations along the basilar membrane. This is accompanied by persistent declines in suprathreshold amplitudes of neural, but not outer hair cell (OHC), responses (2, 5, 23, 24). Here, we aimed to determine whether Trk agonist treatment could ameliorate some of the irreversible damage and reduce neural function caused by this noise exposure.

**Figure 2:**
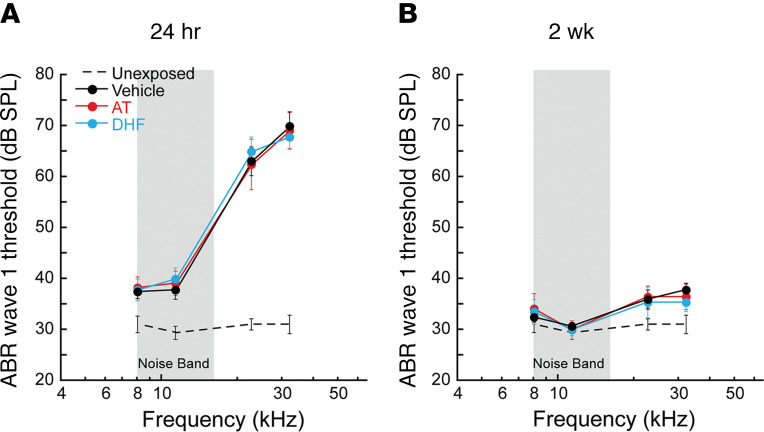
Noise-induced threshold elevations were similar across groups, and Trk agonists did not alter recovery. (**A**) Wave 1 thresholds recorded 24 hours after noise (8–16 kHz, 100 dB SPL, 2 hours; gray bar) and before delivery of drugs in AP, or the control AP solution alone to the posterior semicircular canal, were similar across treatment groups (AT, 25 mM; DHF, 5 mM; AP vehicle). (**B**) By 2 weeks after noise, all groups recovered similarly. Data are shown as mean ± SEM; *n* = 6–8/group. In both panels, thresholds for 16-week unexposed CBA/CaJ mice (23) are included for comparison.

We reasoned that direct administration of the TrkB agonists to perilymph would be the best route for administration of the drugs in vivo. We delivered AT, DHF, or vehicle alone into perilymph via posterior semicircular canal injection 48 hours after noise exposure. When tested 24 hours after exposure (before drug treatment), animals displayed similar ABR wave 1 threshold elevations at test frequencies above the noise band (21–45 kHz, Figure 2A). Thresholds recovered to within about 5 dB by 2 weeks (Figure 2B). Results are consistent with our previous reports (2, 23) and demonstrate, further, that the drug treatments did not alter threshold recovery after noise.

**Figure 3:**
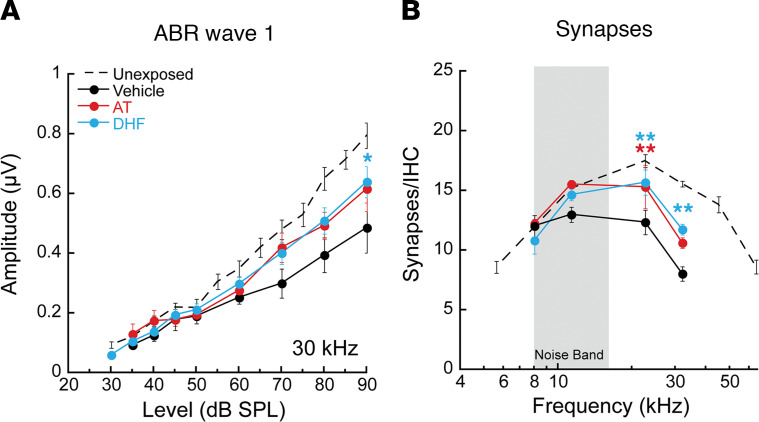
Local, postexposure treatment with Trk agonists recovers cochlear nerve function and IHC synapses. (**A** and **B**) Animals treated with 25 mM AT or 5 mM DHF 48 hours after noise showed significantly greater ABR wave 1 amplitudes (**A**; shown, for example, at 30 kHz; F_2, 130_ = 4.636, *P* = 0.0114) and synapse counts (**B**; F_2, 72_ = 8.182, *P* = 0.0006) than controls treated with vehicle alone. Data are shown as means ± SEM, *n* = 6–8/group. Results of 2-way ANOVA comparing vehicle-only controls and drug-treated animals, with Bonferroni multiple comparisons test, are indicated as follows: *P < 0.05, **P < 0.01. ABR wave 1 response amplitudes and synapse counts from untreated mice (23) are included for comparison.

In basal cochlear regions of maximum noise injury, both AT- and DHF-treated mice demonstrated significantly greater neural response amplitudes than control mice treated with the vehicle alone when assessed at the 2-week postexposure time point (Figure 3A; 2-way ANOVA, F_2, 130_ = 4.636, *P* = 0.0114). Proportionately, and in the same noise-damaged cochlear region, AT- and DHF-treated mice demonstrated significantly greater synapse counts than control mice treated with vehicle alone (Figure 3B; 2-way ANOVA, F_2, 72_ = 8.182, *P* = 0.0006). Two weeks following exposure, ABR wave 1 amplitude and synaptic declines of almost 50% were observed in the noise-damaged basal cochlea of vehicle-treated mice, whereas corresponding values recorded in AT- and DHF-treated animals were at 70%–75% of unexposed mice.

### AT preserved synapses and auditory function in vivo when administered systemically prior to noise exposure.

Given the effects of directly administered drug, and the potential to give these drugs systemically due to their low-risk safety profile, we next asked whether the same effects could be achieved by systemic delivery. We performed these experiments with AT.

**Figure 4:**
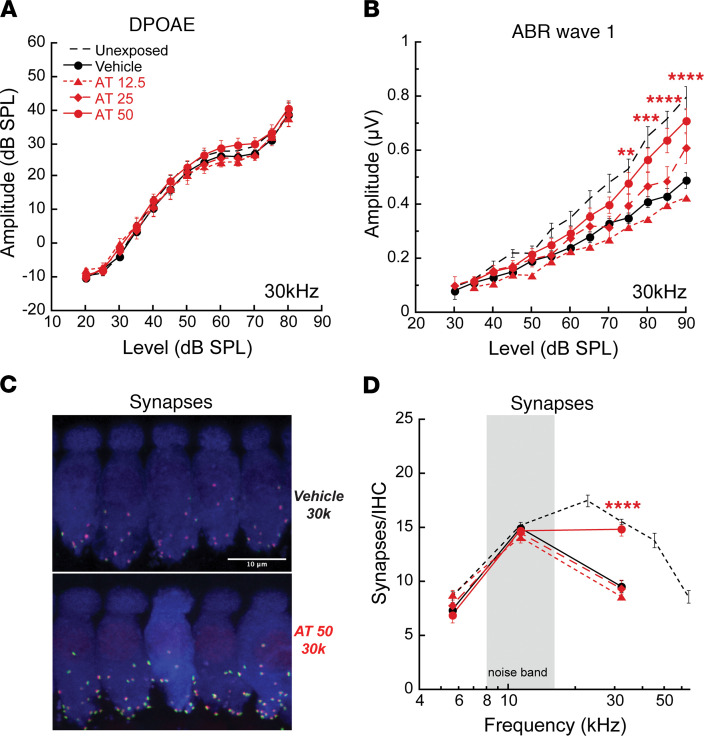
Systemic AT protection is dose responsive. (**A** and **B**) Amitriptyline in saline (AT; 12.5, 25, or 50 mg/kg) or the saline vehicle alone was delivered once daily for 5–9 days. Animals were noise exposed on day 3, 6 hours after injection. DPOAE amplitudes recovered by 2 weeks, but ABR amplitudes did not (compare **A** and **B**). Compared with vehicle-only controls, a significant preservation of ABR wave 1 amplitude at 30 kHz (**B**) (F_1, 392_ = 70.39, *P* < 0.0001) is apparent in mice treated with 50 mg/kg AT. (**C** and **D**) Similarly, synapses (pictured in **C** and quantified in **D**) show significant protection by high-dose AT in the 30 kHz region relative to vehicle-only controls (F_1, 116_ = 18.90, *P* < 0.0001). Data are shown as mean ± SEM, *n* = 10–30/group). (**A**, **B**, and **D**) Results of 2-way ANOVA comparing vehicle-only controls and drug-treated animals, with Bonferroni multiple comparisons test; **P < 0.01, ***P < 0.001, ****P < 0.0001. Untreated mice (23) are shown for comparison.

In our initial experiments, mice were treated systemically with saline or AT in saline (12.5, 25, or 50 mg/kg) before and after noise exposure. Subsets of animals were tested 24 hours after exposure and displayed expected and similar threshold elevations for both DPOAEs and ABR wave 1 at test frequencies above the noise band. Thresholds recovered by 2 weeks, as shown previously for this exposure (2, 23). DPOAE amplitudes at 2 weeks showed essentially complete postexposure recovery for all drug and saline treatment groups (Figure 4A). Together with the DPOAE and ABR wave 1 threshold recovery, the finding is consistent with functionally intact OHCs and no effect of the drugs on their postexposure recovery.

In contrast, 2 weeks after noise, animals showed permanent ABR wave 1 amplitude decrements that varied with treatment and dose (Figure 4B). At the highest dose of AT, 50 mg/kg, response amplitudes averaged over 80–90 dB were significantly larger (2-way ANOVA, F_3, 238_ = 11.07, *P* < 0.0001; Bonferroni multiple comparison significance at 17 kHz [*P* < 0.03] and 30 kHz [*P* < 0.001]) than those recorded for animals in the other groups. Synapse counts at cochlear regions spanning the frequency regions assessed physiologically also were greater in drug-treated animals (2-way ANOVA, F_1, 116_ = 18.90, *P* < 0.0001) (Figure 4, C and D). Vehicle-treated mice showed a maximum 50% synapse loss relative to unexposed ears, whereas counts were nearly normal for AT 50 mg/kg–treated mice.

Subsequent experiments focused on the effective dose (50 mg/kg) and compared outcomes for treatments given only before or only after noise, as follows: (a) once, 6 hours before noise; (b) once, 3 days after noise; and (c) once daily for 9 days beginning 3 days after exposure.

**Figure 5:**
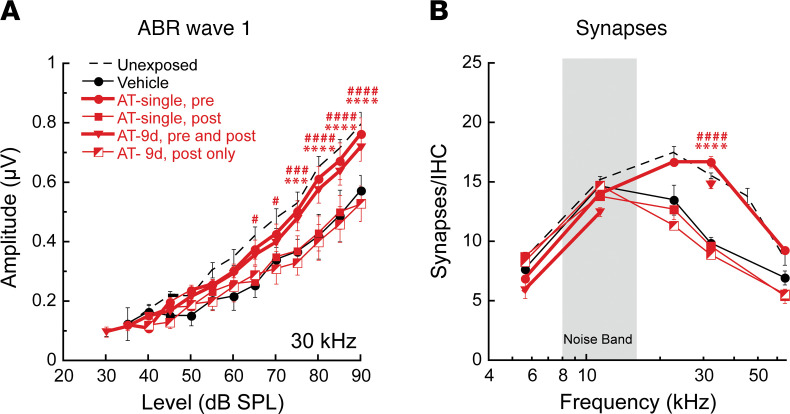
Systemic AT, delivered before but not after exposure, protects cochlear nerve function and IHC synapses. (**A** and **B**) Groups of animals received i.p. injections of AT in saline or saline vehicle alone. Shown are data for (a) AT (50 mg/kg) or saline once, 6 hours prior to noise exposure; (b) AT (50 mg/kg) or saline once, 3 days after noise exposure; (c) AT (50 mg/kg) or saline before and after noise (once daily for 9 days with noise exposure delivered 6 hours after the third day of treatment); and (d) AT (50 mg/kg) or saline solely after noise (once daily for 9 days beginning 3 days after noise exposure). Together, results show systemic AT required preexposure delivery (groups with heavy lines) to achieve protection (ABR amplitude, F_2, 496_ = 55.49, *P* < 0.0001; synapses, F_2, 143_ = 24.19, *P* < 0.0001). Data are shown as mean ± SEM, *n* = 10–30/group. Results of 2-way ANOVA with Bonferroni multiple comparisons test are indicated as follows: *P < 0.05, **P < 0.01, ***P < 0.001, ****P < 0.0001 for “AT-single, pre” condition; ^#^*P* < 0.05, ^##^*P* < 0.01, ^###^*P* < 0.001, ^####^*P* < 0.0001 for “AT-9d, pre and post” condition. For comparison, counts were from for 16-week unexposed CBA/CaJ mice (23).

Postexposure threshold recovery by 2 weeks was again unaltered by the treatments. However, wave 1 amplitudes and synapse counts at basal cochlear frequencies were sensitive to the timing of treatment relative to noise exposure; both were near normal when AT was given before noise but declined to approximately 50% when AT was administered only after noise (Figure 5, A and B). For animals receiving single-dose prenoise AT, suprathreshold wave 1 amplitudes and IHC synapse counts at 2 weeks were approximately 50%–60% larger than saline controls in the noise-damage region (17 and 30 kHz). However, when the same AT dose was delivered 6 hours after noise, neither ABR wave 1 amplitudes (Figure 5A) nor synapse counts (Figure 5B) were systematically different from those recorded for saline controls (amplitudes, F_1,__58_ = 3.742, *P* = 0.0579; synapses: F_1, 112_ = 0.06407, *P* = 0.8006; values given here are for those conditions not reaching significance). Thus, AT showed potent protection but only at the highest dose (50 mg/kg), and a single injection at high concentration was as effective as repeated injections.

### AT preserved synapses and auditory function in vivo 1 year after noise exposure.

**Figure 6:**
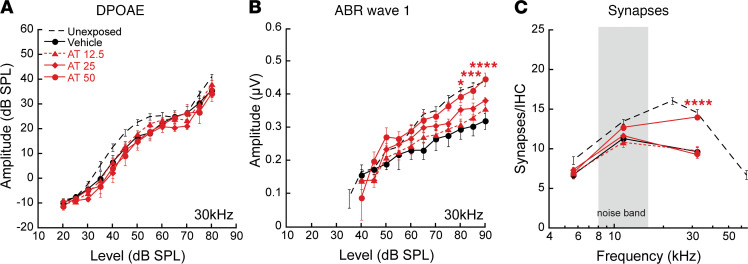
AT protection is long-lasting. (**A**) Subsets of mice were held 1 year after noise. DPOAE amplitudes remained well preserved in all groups. (**B**) Relative to age-matched, vehicle-treated controls, significant conservation of ABR wave 1 amplitude was observed in mice treated with 50 mg/kg AT for 9 days beginning 3 days before noise (F_1, 122_ = 33.88, *P* < 0.0001). (**C**) The number of synapses remaining at 2 weeks was also maintained 1 year after noise exposure, with significantly more synapses in the 30 kHz region for mice treated with 50 mg/kg AT (F_1, 131_ = 16.71, *P* < 0.0001). Data are represented as ± SEM; *n* = 7–30/group. Results of 2-way ANOVA with Bonferroni multiple comparisons test are shown; *P < 0.05, ***P < 0.001, ****P < 0.0001. Unexposed, age-matched animals (23) are shown for comparison.

Prior work has documented gradually progressive loss of synapses with aging and acceleration of these losses with spread to more apical regions in animals receiving synaptopathic noise exposure as young adults (5, 23). Here, subsets of animals from the initial experimental series (systemic AT 12, 25, 50 mg/kg) were followed to 1 year after exposure to assess long-term effects of drug treatments on cochlear deafferentation. Shown in Figure 6, A and B, are DPOAE and ABR wave 1 amplitudes (30 kHz) for these groups. OHC-based DPOAEs are similar across treated groups, documenting minimal exacerbation of ongoing declines relative to age-only mice (23). Of note, the neuroprotective treatment effect of AT apparent at 2 weeks (Figure 4C) persists, with mice that received prenoise AT (50 mg/kg) displaying significantly greater neural response amplitudes at 17 kHz (*P* < 0.01) and 30 kHz (*P* < 0.05) than vehicle-treated animals at the 1-year test time (2-way ANOVA, F_1, 69_ = 11.46, *P* < 0.01 with Bonferroni multiple comparisons test). Indeed, with responses little different from never-exposed mice, these animals appear to have been fully protected from the noise-induced cochlear deafferentation.

Support for this notion also can be found in the synapse counts from these mice (Figure 6C). High-dose AT-treated mice showed significantly greater (F_1, 131_ = 16.71, *P* < 0.0001) synapse counts than vehicle-treated ears at 30 kHz (*P* < 0.0001), where counts were consistent with age-matched, unexposed mice.

Moreover, vehicle-treated animals that were noise exposed, then aged, showed greater exacerbation of losses in the apical region that otherwise occur with aging than did AT-treated mice that were noise exposed and held identically. This can be seen in both ABR wave 1 amplitude and synapse data by comparing Figure 5 (animals 2 weeks after exposure at 16 weeks) and Figure 6 (animals 1 year after exposure at 16 weeks).

When compared with data collected for animals held 2 weeks following noise exposure, wave 1 amplitude differences between vehicle- and AT-treated mice were exaggerated at 1 year, with vehicle-treated animals showing greater ongoing declines after noise. Of note, DPOAE amplitudes recorded from the same ears were comparable for all groups. Together, findings show that drug-related effects target synapses/neurons rather than OHCs, effects are long-lasting, and the increasing separation between saline and AT (50 mg/kg) is not reflected at the level of the OHCs. Additionally, as synapse counts in saline-treated animals show results consistent with our previous reports in exposed, then aged mice (23), the significantly smaller age-progressive declines for AT-treated mice suggest that the drug also may have a long-term protective effect against age-progressive deafferentation.

## Discussion

We have shown here that treatment with AT restores synapses between cochlear afferent neurons and sensory IHCs. In contrast to the ears of control (vehicle-treated, noise-exposed) animals, in which extensive noise-induced afferent synapse loss persists after thresholds recover, synapses were significantly protected by preexposure treatment and rescued by postexposure treatment with AT, and cochlear neurons appeared functionally intact. The protective effect of prenoise AT against cochlear deafferentation was long-lasting. The regenerative effect of the drug is particularly significant for clinical application, as exposure to noise, which may be a principal contributor to synaptopathy in the human population, can occur in varied environments with unpredictable timing and may be cumulative. The effect of AT in mitigating the synaptic loss and promoting synaptogenesis was also seen in vitro, providing support for a regenerative mechanism and pointing to TrkB receptor involvement.

Synaptopathy occurs as a primary consequence of noise exposure and can permanently reduce cochlear afferent response amplitudes in ears with functionally intact hair cells and normal thresholds (2). Correlation between loss of amplitude and synaptic loss suggested a “hidden hearing loss” in which audiograms (assays of threshold sensitivity) could recover to normal and the loss of synapses preceded the loss of hair cells and could act independently of it. However, even when thresholds recover, this cochlear deafferentation may compromise the fidelity of suprathreshold signal coding and, moreover, appears to be a harbinger of exaggerated declines in hearing function (1, 2). Indeed, this type of mechanism for loss of neural response amplitude has now been shown to be important in the loss of cochlear function caused by aging, as well as noise exposure (5).

The activity we find here for a Trk agonist is consistent with previous data on Trk receptor activation by Trk ligands NT3 and BDNF (4, 9–12). NT3 and BDNF have long been shown to be protective or ameliorative for neural loss in the cochlea. The roles of NT3 and BDNF may be different in synaptogenesis, fiber growth, and survival. The latter roles, neuron survival and growth, are the most thoroughly characterized of the effects and are found throughout the nervous system. NT3 and BDNF do not appear to be equivalent for these roles (25–27). Both neurotrophins are present in the inner ear, and several studies have shown that NT3 is the principal neurotrophin in the cochlea, whereas BDNF has a more important role in the vestibular system (28). However, there is considerable overlap of function. Indeed, the 2 neurotrophins have been used together in many studies (12), and both stimulate neurite outgrowth and neuron survival.

Neurotrophins also show distinct actions in regeneration of neurons. This distinction may be key to the effects of AT on regeneration versus protection. AT has been shown to have TrkB activity with no TrkC activity (14). There are some indications that NT3, in particular, is effective in cochlear hearing loss, whereas BDNF is effective in vestibular dysfunction due to synaptic lesions (29–31). NT3 but not BDNF appeared to reverse the synaptopathy in a genetic model (31), and NT3 application to the round window (30) protected synapses from noise-induced synaptopathy. If indeed NT3 stimulation of TrkC but not BDNF stimulation of TrkB can repair cochlear synapses, then it seems logical that a TrkB agonist would be suboptimal. Since we see a protective effect when the drug is given prior to noise exposure, as well as a regenerative effect when the drug is given after noise exposure, we believe that TrkB stimulation is effective for the stimulation of synaptogenesis in the cochlea. The effect of AT suggests that TrkB agonist activity is sufficient to restore synapses. It is difficult to compare our data with the results of BDNF induction in transgenics, where the concentration of peptide is unknown and may differ from the BDNF equivalents we added. However, we cannot rule out the possibility that high concentrations of the drug could also produce some activation of TrkC.

BDNF has a stimulatory effect on regeneration in many systems and is released in response to damage. In studies of aminoglycoside-induced hearing loss, ears that were administered neurotrophins after the insult showed less neural loss (9, 11, 12); however, this effect was ascribed to protection from neural loss that occurs secondary to the loss of hair cells. Regeneration of synapses was reported in the newborn rat in vitro (20). In that case, the synapses partially recovered spontaneously, and the recovery could be augmented by BDNF or NT3. However, the endogenous factor responsible for the spontaneous regeneration appeared to be NT3. Protection of hearing from noise exposure was correlated to secretion of BDNF that occurred at night but not during the day (32). In the studies reported here, administration of AT after the noise exposure restored synapses when it was at sufficient concentrations. Posterior semicircular canal delivery proved the most effective route and was presumed to be successful because the drug reached the cochlear perilymph at sufficient concentrations. Given the regenerative effect of AT on cochlear neurons, the possibility remains that the observed protective effect was also a regenerative effect occurring after the noise exposure (i.e., a fast regeneration comprising synaptic remodeling on a rapid time scale as occurs in synaptic spines in a process driven by BDNF; ref. 33). The time window we looked at for administration of the drug was limited to the period shortly (2 days) after noise exposure. Development of a regenerative treatment using this drug will require assessment of the full extent of the postdamage window for a therapeutic effect.

Tricyclic antidepressants like AT have Trk agonist activity (14). Interestingly, this activity was discovered long after their introduction for the treatment of mood disorders. The compounds were initially used for inhibition of serotonin and noradrenaline transport (34–36). Since the discovery of the neurotrophic activity, the compounds have been tested for the amelioration of various types of hearing loss (15–17). The activity on a genetic hearing disorder was striking (17) and was thought to mimic BDNF activity. The effect on noise damage was thought to be due to protection of SGNs (15). AT is a well-tolerated drug that has been tested thoroughly for mood disorders, and our data suggest that AT or related drugs could have important clinical applications in noise-induced hidden hearing loss as studied here and potentially in age-related hidden hearing loss related to cochlear deafferentation.

## Methods

### Animals.

Mice (CBA/CaJ) were born and raised in our acoustically monitored animal care facility (5) from breeders purchased from the Jackson Laboratory.

### In vitro coculture of neonatal SGNs and deafferented organ of Corti.

In vitro coculture of SGN and deafferented organ of Corti was performed according to the method described previously (19, 37). Briefly, isolated SGNs were treated with 0.25% trypsin for 15 minutes at 37°C. After neutralization of the trypsin with 10% FBS in DMEM, neurons were collected by centrifugation (300*g* for 5 minutes at room temperature), triturated to a single-cell suspension, and cocultured with the deafferented organ of Corti. The organ was dissected by removal of the membranous labyrinth, the stria vascularis, and spiral ligament. Reissner’s membrane and the tectorial membrane were removed, and the organ was cultured in a well on a cover glass coated with 1:1 poly-l-ornithine (0.01%; MilliporeSigma) and laminin (50 μg/mL; BD Biosciences). Explants were cultured in N2- and B27-supplemented DMEM, 1% HEPES solution (MilliporeSigma), 1:1000 ampicillin, 1:300 fungizone at 37°C, and 6.5% CO_2_. AT (0.5 μM; MilliporeSigma) was added at the beginning of the coculture, and cultures were stopped after 6 days. The number of innervated IHCs at the end of the incubation was quantified (37, 38).

### In vitro glutamate toxicity.

Organ of Corti explants from P3–P4 mice of both sexes, after 24 hours in culture, were exposed to a solution of 0.4 mM kainic acid (Abcam) diluted in culture medium. After a 1-hour kainic acid treatment, the explants were washed 6 times with culture medium and maintained for 24 hours in 80 μL of culture medium alone or containing AT (0.1 μM) or DHF (0.5 μM; MilliporeSigma). The cultures were then fixed and prepared for immunofluorescence.

### Acoustic overexposure.

Noise exposure (8–16 kHz octave-band noise, 100 dB SPL, 2 hours) was conducted on awake, adult (16-week) mice as described previously (2). The noise was created by a waveform generator (model WGI; Tucker-Davis Technologies), filtered (8–16 kHz, >60 dB/octave slope; Frequency Devices), amplified (D-75 power amplifier; Crown Audio), and delivered (compression driver; JBL) through an exponential horn into a tabletop reverberant exposure chamber. Mice were placed in a subdivided cage suspended directly beneath the acoustic horn of the sound-delivery speaker. Prior to each exposure, the noise was calibrated to the target 100 dB SPL using a quarter-inch condenser microphone (variability < 1 dB across subdivisions).

### Drugs and delivery.

To optimize drug access to the cochlea, direct infusion of the drug into the posterior semicircular canal was performed. For these experiments, animals that were awake were noise exposed and held for 48 hours. They were then anesthetized (ketamine 100 mg/kg and xylazine 10 mg/kg, i.p.). A small hole was made in the posterior semicircular canal, and 500 nL of drug suspension (25 mM AT in artificial perilymph; or 5 mM DHF in 10% DMSO in artificial perilymph; or artificial perilymph without drug) was injected at a rate of 91 nL/min. At the end of the infusion, the needle was left in place for 5 minutes, after which the surgical defect was repaired. Animals were held for 2 weeks before testing and processing.

To assess effects with less invasive, systemic delivery of drugs, we focused on AT, and animals received i.p. injections of AT in saline or an equal volume of the saline vehicle alone according to the following treatment groups: (a) AT (12.5, 25 or 50 mg/kg) or saline once daily for 5–9 days with noise exposure 6 hours after the third day of treatment; (b) AT (50 mg/kg) or saline once, 6 hours prior to noise exposure; (c) AT (50 mg/kg) or saline once, 3 days after noise exposure; and (d) AT (50 mg/kg) or saline once daily for 9 days beginning 3 days after noise exposure. Groups of mice were evaluated 24 hours after noise to assess acute losses, 2 weeks after noise, when threshold recovery for this exposure is complete and synapse loss has stabilized (2), or were held for 1 year after exposure to examine long-term effects.

### Cochlear function tests.

DPOAEs and ABRs were recorded from anesthetized mice (ketamine 100 mg/kg and xylazine 10 mg/kg, i.p.) in a 37°C, acoustically and electrically shielded chamber. A small incision made in the cartilaginous portion of the external ear canal provided a clear view to evaluate the health of the tympanic membrane and optimized acoustic system placement. Physiologic responses were stimulated and measured using a 24-bit National Instruments PXI–based system controlled by custom LabView-based software. The acoustic system consisted of 2 miniature dynamic earphones (CDMF15008-03A; CUI Devices) and a condenser microphone (FG-23329-PO7; Knowles) coupled to a probe tube.

OHC-based DPOAEs were recorded in response to 2 primary tones at frequencies (f) f1 and f2, with f2 equal to the frequencies used in ABR testing, f2/f1 = 1.2 and levels (L) L2 = L1 – 10 dB. For each frequency pair, DPOAE responses at 2f1 – f2 were recorded, averaged, and analyzed as a function of level (L2, below threshold to 80 dB SPL) from sound pressure measurements in the ear canal. Threshold was defined as the L2 stimulus level required to generate a DPOAE amplitude of –5 dB SPL.

Neural-based ABRs were measured in response to tone pips (5.6–45.2 kHz; 0.5 ms rise-fall, 5 ms duration, 30/s, alternating polarity) using subdermal electrodes placed at the vertex, ventrolateral to the pinna, and at the base of the tail (ground). Stimuli were delivered at subthreshold levels to 90 dB SPL in 5 dB steps. Amplified (10,000×) responses were filtered (0.3–3 kHz), digitized, and averaged for each of the frequency-level combinations (1024 samples/level). Threshold was identified via visual inspection of stored waveforms as the lowest stimulus level needed to elicit a repeatable wave 1 response.

### IHC and quantification of afferent synapses.

Immediately following the physiologic testing, anesthetized mice were transcardially perfused with 4% paraformaldehyde in 0.1 M phosphate buffer with additional perfusion through the cochlear scalae. Cochleas were postfixed in 4% paraformaldehyde for 1 hour and then decalcified (0.12 M EDTA) for up to 48 hours. Microdissected pieces were placed in a blocking buffer (PBS with 5% normal horse serum and 0.3% Triton X-100) for 1 hour at room temperature, followed by overnight incubation at 37°C in antibodies to the following: (a) C-terminal binding protein 2 (mouse anti-CtBP2; BD Biosciences, catalog 612044; used at 1:200); (b) myosin-VIIa (rabbit anti–myosin-VIIa; Proteus Biosciences, catalog 25-6790; used at 1:200); and (c) GluA2 (mouse anti–glutamate receptor 2; MilliporeSigma, catalog MAB397; used at 1:2000). Cochlear pieces were incubated in species-appropriate secondary antibodies coupled to Alexa Fluor in the red, blue, and green channels for 2 hours at 37°C.

Cochlear piece lengths, collected and compiled using ImageJ (NIH) software, were used to create a cochlear frequency map to localize cochlear structures to specific frequency regions (39). Confocal *Z*-stacks were obtained (Leica TCS SP5) using a high-resolution, glycerin-immersion objective (63×, 1.3 NA) and a 3.17× digital zoom, 1024 × 512 raster. Two adjacent stacks (0.25 μm step size) were imaged for each targeted frequency region, each image spanning 78 μm of cochlear length. Confocal image stacks were ported to image-processing software (Amira; VISAGE Imaging) where IHCs and synapses were quantified as previously described (5, 23). Synaptic associations between presynaptic IHC ribbons and postsynaptic glutamate receptors (synapses) were determined with custom software that computed and displayed the x-y projections of the voxel space within 1 μm of the center of each ribbon identified by Amira analysis (23).

IHC and quantification of regenerated afferent synapses in the in vitro assays were performed by the same methods (19). Following fixation and permeabilization for 1 hour, cultures were incubated with primary antibodies, anti-CtBP2 (mouse anti-CPBP2, BD Biosciences, catalog 612044; used at 1:1000), anti–Psd95 (mouse anti–Psd95; NeuroMab, clone K28/43; used at 1:1000), and anti-neurofilament (chicken antibody to neurofilament-H; MilliporeSigma, catalog AB5539; used at 1:2500) overnight at 4°C. After rinsing 3 times for 10 minutes with 0.01 M PBS, pH 7.4, they were incubated with secondary antibodies: Alexa Fluor 633 goat anti–mouse IgG1 (Thermo Fisher Scientific, catalog A-21126), biotin-conjugated Alexa Fluor goat anti–mouse IgG2a (Thermo Fisher Scientific, catalog M32315), Alexa Fluor 568–Streptavidin (Thermo Fisher Scientific, catalog S11226), and Alexa Fluor 488 goat anti-chicken (Thermo Fisher Scientific, catalog A-11039) for 2 hours at room temperature. Newly generated afferent synapses were identified by triple labeling of CtBP2, Psd95, and neurofilament.

### Statistics.

Statistical analyses were conducted using Prism 7.0 software (GraphPad). For analyses on adult mice, a 2-way ANOVA was used, followed by Bonferroni correction for multiple comparisons. For cocultures of neurons and the organ of Corti and kainate-treated organ of Corti, results were analyzed by a 2-tailed, unpaired Student’s *t* test. For all analyses, α < 0.05 was considered significant.

### Study approval.

All experiments were conducted in accordance with the Public Health Service policy on Humane Care and Use of Laboratory Animals.

## Author contributions

SGK and ASBE acquired funding; KAF, TW, MT, XM, KT, SGK, and ASBE designed research; KAF, TW, MT, XM, and KT performed research; KAF, TW, SGK, and ASBE analyzed data; and KAF, SGK, and ASBE wrote the paper.

## Version 1 12/29/2020

In-Press Preview

## Version 2 02/08/2021

Electronic publication
